# A Comprehensive Review of Applications and Environmental Risks of Waste Plastics in Asphalt Pavements

**DOI:** 10.3390/ma18153441

**Published:** 2025-07-22

**Authors:** Ju Pan, Jue Li, Bailin Shan, Yongsheng Yao, Chao Huang

**Affiliations:** 1 School of Traffic & Transportation, Chongqing Jiaotong University, Chongqing 400074, China; pan_ju2024@163.com (J.P.); bl.shan@cqjtu.edu.cn (B.S.); yaoyongsheng23@cqjtu.edu.cn (Y.Y.); 2 School of Civil Engineering, Central South University, Changsha 410075, China; huangchao-22@csu.edu.cn

**Keywords:** waste plastics, microplastic pollution, toxic emissions, sustainable pavement

## Abstract

The global plastic crisis has generated significant interest in repurposing waste plastics as asphalt modifiers, presenting both environmental and engineering advantages. This study offers a comprehensive review of the applications of waste plastics in asphalt, focusing on their types, modification mechanisms, incorporation techniques, and environmental impacts, alongside proposed mitigation strategies. Commonly utilized plastics include polyethylene (PE), polypropylene (PP), polystyrene (PS), polyvinyl chloride (PVC), and polyethylene terephthalate (PET), each affecting asphalt performance differently—enhancing high-temperature stability and fatigue resistance while exhibiting varying levels of compatibility and environmental risks. The incorporation techniques, namely wet and dry processes, differ in terms of efficiency, cost, and environmental footprint: the wet process enhances durability but requires more energy, whereas the dry process is more cost-effective but may lead to uneven dispersion. Environmental concerns associated with these practices include toxic emissions (such as polycyclic aromatic hydrocarbons and volatile organic compounds) during production, microplastic generation through abrasion and weathering, and ecological contamination of soil and water. Mitigation strategies encompass optimizing plastic selection, improving pre-treatment and compatibilization methods, controlling high-temperature processing, and monitoring the spread of microplastics. This review highlights the need for balanced adoption of waste plastic-modified asphalt, emphasizing sustainable practices to maximize benefits while minimizing risks.

## 1. Introduction

The global plastic crisis constitutes a critical environmental challenge that necessitates urgent action. Over the past seventy years, global plastic production has escalated dramatically from 2 million metric tons in 1950 to 368 million metric tons in 2018, with forecasts indicating it may surpass 500 million tons by 2025 [1]. This rapid increase has profoundly reshaped lifestyles and consumption patterns, integrating plastics into the fabric of modern society. However, the inadequacy of waste management systems has intensified the environmental crisis, as only 9% of all plastic waste generated has been recycled and 12% incinerated, leaving a staggering 79% to accumulate in landfills or the natural environment [2,3].

The persistence of plastic waste in the environment poses significant long-term threats to ecosystems. Its resistance to natural degradation contributes to the proliferation of microplastics, landfill overflow, and contamination of both terrestrial and aquatic ecosystems [4,5]. Microplastics, defined as plastic particles smaller than 5 mm, have emerged as widespread environmental pollutants [6]. These particles can originate from the degradation of larger plastic items or be intentionally produced for various applications, including cosmetics and cleaning products. The ubiquitous presence of microplastics, found in environments ranging from oceanic trenches to mountain peaks, poses substantial risks to ecosystem health and resident organism [7].

In light of these pressing challenges, the quest for effective strategies to manage and repurpose plastic waste has become increasingly critical. In cementitious materials, waste plastics are often used as reinforcing agents to enhance mechanical properties. For example, the incorporation of recycled plastic mesh has been shown to improve the flexural toughness and ductility of mortar beams [8]. Similarly, recycled plastic fibers can enhance the flexural toughness and reduce plastic shrinkage cracking in cement mortar [9]. However, the integration of waste plastics into cementitious materials faces challenges, including suboptimal interfacial bonding and potential reductions in durability [10]. Given the widespread use of asphalt pavements, the incorporation of waste plastics into asphalt has also gained significant attention [11]. Traditional asphalt modification materials, including virgin polymers and rubber, face limitations such as high production costs and significant natural resource consumption [12]. The utilization of waste plastics as asphalt modifiers has the potential to enhance properties such as aging resistance, cracking resistance, and high-temperature performance, while simultaneously reducing petroleum consumption and construction costs [13,14,15].

Despite the advantages offered by waste plastic integration into asphalt, several challenges persist. Emerging research highlights various environmental and health concerns associated with this practice [16,17]. For instance, the volatilization of plastic components at elevated temperatures may release toxic gases, thereby compromising air quality [18]. Moreover, harmful substances, such as heavy metals and plasticizers found in waste plastics, may leach into soil and groundwater throughout the lifespan of the asphalt, posing significant pollution risks [19]. Pollution from waste plastics can adversely affect public health through pathways such as inhalation, dermal contact, contaminated drinking water, and the food chain, due to the close relationship between environmental and human health [20,21]. These concerns underscore the necessity for a thorough understanding of the associated trade-offs and the development of effective mitigation strategies.

This paper provides a comprehensive overview of the applications of waste plastics in asphalt, along with the associated environmental effects, with attention to both ecological impacts and potential threats to human health. The overview begins by exploring the types of waste plastics commonly used in asphalt applications and the various incorporation techniques, such as wet and dry processes. It then goes on to discuss the environmental concerns linked to the use of waste plastics in asphalt, focusing on toxic emissions during production, the generation of microplastics, and the possibility of ecological contamination. A detailed assessment of human health risks, including both direct exposure and indirect threats, is also provided. Finally, the overview presents mitigation strategies and sustainable solutions to tackle the identified challenges.

## 2. Waste Plastic Types and Modification Effects

### 2.1. Classification and Properties of Waste Plastics

**Figure 1:**
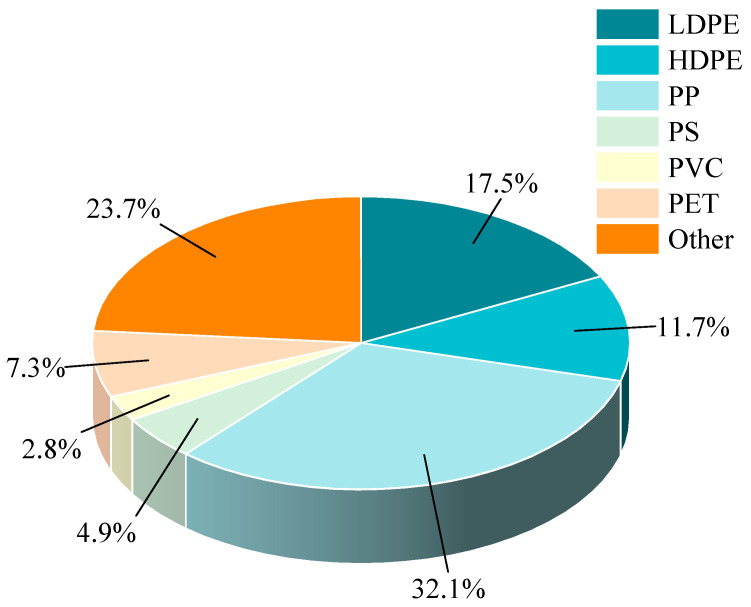
Breakdown of plastics in MSW.

Plastics are organic polymer materials composed of large organic molecules, primarily derived from various sources, including household waste, industrial byproducts, agricultural residues, and commercial packaging discards [22]. The breakdown of plastic content within municipal solid waste (MSW) is illustrated in Figure 1, which underscores the substantial contribution of plastics to overall waste generation [23]. A significant proportion of discarded plastics, including polyethylene (PE), polypropylene (PP), polystyrene (PS), and polyvinyl chloride (PVC), can be attributed to the relatively short lifespan of products made from these materials, often lasting less than two years [24].

The incorporation of waste plastics into asphalt pavement systems involves a diverse array of plastic types, each possessing distinct physicochemical properties that can significantly influence the performance and processability of the modified asphalt. The primary plastic categories commonly utilized in asphalt applications include high-density polyethylene (HDPE), low-density polyethylene (LDPE), PP, PS, PVC, and polyethylene terephthalate (PET).

Table 1 summarizes the fundamental properties of several major waste plastics, including their melting points, primary sources, mechanical properties, and potential environmental impacts. Each type of plastic exhibits unique characteristics that influence its suitability for asphalt modifications. For example, LDPE exhibits high flexibility, which enhances the fatigue crack resistance of asphalt. In contrast, PP, with its moderate tensile strength, is preferred in environments exposed to chemical agents due to its superior chemical resistance. However, PS poses challenges due to its limited recyclability and high rigidity, which may compromise asphalt performance.

The environmental impacts of these plastics underscore the need for careful selection and lifecycle management when incorporating waste plastics into asphalt. While plastics like HDPE are highly recyclable and have reduced environmental impacts, others, such as PS and PVC, pose significant risks through microplastic formation and the release of toxic additives.

### 2.2. Modification Mechanisms and Effects

The characteristics of different types of waste plastics can vary significantly due to differences in their chemical composition, molecular structure, and average molecular weight [37]. These variations directly influence the modification mechanisms and resultant performance effects of waste plastics when incorporated into asphalt. A comparative analysis of the modification effects of various common waste plastics on asphalt is presented in Table 2. Based on the table, it can be concluded that incorporation of waste plastic generally enhances the high-temperature stability and fatigue resistance of modified asphalt. In addition, EVA and LDPE show superior overall performance but may be less cost-effective for low-traffic roads.

LDPE features long, flexible linear chains that can interlock with the branched structures present in asphalt, forming a three-dimensional network. Consequently, LDPE demonstrates superior compatibility with asphalt compared to other polyethylene types [38]. Studies have demonstrated that modified asphalt binders containing 10% LDPE show optimal rutting resistance relative to other modifiers [39]. The fatigue life (Nf) indicates that LDPE-modified asphalt enhances fatigue resistance compared to unmodified asphalt [41]. However, it is important to note that the low-temperature performance of the modified asphalt decreases as the LDPE dosage increases [40].

HDPE-modified blends exhibit increased stiffness, which contributes to improved rutting resistance [42]. The appropriate proportion of HDPE can significantly extend pavement life and reduce maintenance frequency [43]. Furthermore, the monotonic cracking index (MCI) suggests that the addition of HDPE in conjunction with PP may increase the risk of low-temperature cracking in the modified bitumen [45].

PP is another commonly used plastic in asphalt modification, characterized by a moderate melting point ranging from 160 °C to 170 °C. This property facilitates melting and uniform dispersion during asphalt mixing. PP’s compatibility with asphalt enhances pavement stability and durability, as evidenced by improved aging index values for PP-modified asphalt [46]. Additionally, PP exhibits optimal flow characteristics at elevated temperatures, improving the high-temperature stability of asphalt [47].

PS has a high melting point (230–240 °C), which presents challenges for complete melting at typical asphalt processing temperatures (150–180 °C). Consequently, PS usually exists in a solid or semi-molten state during mixing. Nonetheless, its rigid structure can enhance the high-temperature performance of asphalt by increasing compressive strength and hardness [48]. Studies have shown that the addition of PS significantly reduces the plastic deformation of asphalt concrete mixtures [49]. However, this enhancement in high-temperature performance increases brittleness, thereby reducing the low-temperature crack resistance of the modified asphalt. Furthermore, the unstable behavior of PS during the mixing process can negatively affect its fatigue resistance [50].

PVC is widely used in various consumer products, but its application in asphalt poses specific challenges. PVC is thermally unstable, and processing it at high temperatures can release hazardous HCl [33]. Consequently, PVC is less frequently employed due to its thermal sensitivity and complex polymer chain structure, which adversely affects compatibility with asphalt. Nevertheless, some studies indicated that PVC can enhance the rutting resistance, low-temperature crack resistance, and fatigue resistance of asphalt binders [51,52,53].

PET, commonly found in beverage bottles and other packaging materials, has also been investigated as a waste plastic additive for asphalt. PET has a high melting point (approximately 250 °C) and high stiffness, which may impair compatibility and low-temperature performance [54]. However, Modarres and Hamedi [55] indicated that PET enhances the fatigue performance of asphalt mixtures at both 5 °C and 20 °C. Additionally, wet PET-modified blends exhibited superior rutting resistance compared to unmodified control blends in indirect tensile strength tests [56].

In addition to the plastics, EVA has garnered attention due to its effective modification effects. The flexibility and elasticity of EVA make it effective in mitigating cracking under low-temperature conditions [57]. Its rigid three-dimensional network structure and polar functional groups ensure excellent compatibility and high-temperature stability in asphalt mixtures [58]. Furthermore, the addition of EVA significantly improves the fatigue resistance of modified asphalt [59].

Overall, the modification mechanisms of these waste plastics can be attributed to their ability to interact with the asphalt matrix at the molecular level. For example, the long-chain structure of LDPE facilitates effective interlocking with asphalt components, enhancing the overall cohesion and structural integrity of the modified asphalt. Similarly, the chemical structure of PP contributes to the formation of a homogeneous blend, which improves the mechanical properties of the asphalt.

Incorporating waste plastics into asphalt enhances performance while addressing environmental concerns by diverting plastic waste from landfills and reducing reliance on virgin materials. However, it is essential to balance these benefits against the potential risks associated with specific plastic types, particularly concerning their long-term behavior and environmental impact.

## 3. Comparison of Modification Techniques and Properties

**Figure 2:**
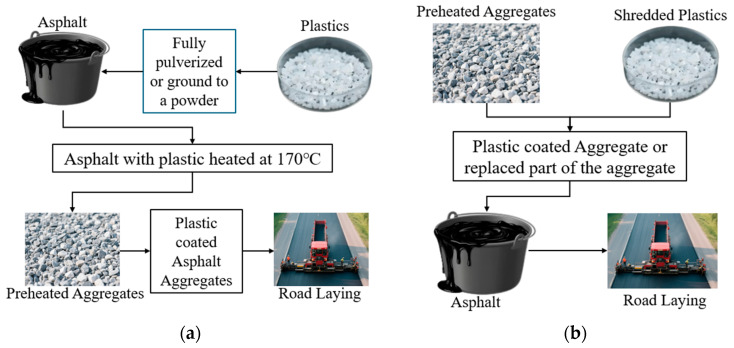
Illustration of (**a**) wet mixing process and (**b**) dry mixing process.

The incorporation of waste plastics into asphalt can be achieved through two primary application techniques: the wet process and the dry process [60], as shown in Figure 2.

In the wet process, waste plastics are first melted and blended with asphalt at high temperatures to form a homogeneous asphalt–plastic mixture, which is subsequently combined with aggregates [61]. This method ensures thorough integration of plastics with asphalt and is suitable for plastics that are soluble in specific solvents or compatible with asphalt, improving the mixture’s stability and compatibility [62]. Failure to achieve proper integration can result in a non-uniform asphalt mixture that lacks cohesion and ductility [63]. In contrast, the dry process involves adding waste plastics directly to hot aggregates, where they partially melt or soften, coating the aggregate surfaces before mixing with asphalt [61]. The plastics selected for this technique typically have high melting points (>180 °C) and thermal stability to maintain structural integrity during mixing [63].

The wet process is widely adopted due to its ability to produce modified mixtures that exhibit reduced fatigue, extended service life, and greater resistance to permanent deformation [64,65,66]. However, the wet process involves a complex processing procedure requiring specialized equipment, which increases costs and raises concerns about the long-term storage stability of the modified asphalt [50,67]. In contrast, the dry process is simpler and potentially more cost-effective, as it eliminates the need for pre-melting and blending the plastic with the asphalt binder. Previous studies indicate that dry-process modified asphalt exhibits improved Marshall stability, indirect tensile strength (ITS) values, and tensile strength ratio (TSR), although its fatigue resistance can be influenced by the type of plastic used [68,69,70]. Nevertheless, the dry process faces challenges, particularly with the uneven dispersion and bonding of plastic particles in the asphalt mixture [71], which can affect the long-term durability and performance of the asphalt [72].

Furthermore, a comparative analysis of the environmental trade-offs between the wet and dry processes reveals distinct advantages and limitations for each technique [73]. The wet process may require more energy and resources due to the melting and blending steps, potentially increasing its environmental footprint [74]. Quantitative evaluations demonstrate that the dry process for producing asphalt mixtures exhibits significant energy-saving benefits compared to the wet process [75]. Recent studies indicate that plastics incorporated via the wet process are more susceptible to microplastic degradation because they are located in the thin asphalt membrane on the pavement surface [76]. Additionally, the dry process may generate more dust and airborne particles, necessitating management strategies to minimize environmental impact [77]

The choice between the wet and dry processes should be based on a comprehensive evaluation of the available production equipment, performance indicators, the source of plastic waste, and the desired properties of the asphalt mixture. Table 3 provides a comparison of various aspects of the wet and dry processes to facilitate decision-making for researchers.

## 4. Environmental Impact Assessment

### 4.1. Toxic Emissions During Production

The incorporation of waste plastics into asphalt pavement systems raises significant concerns about the potential release of toxic emissions during the production and application processes. The high temperatures and processing conditions involved in the manufacturing of waste plastic-modified asphalt can lead to the volatilization and release of hazardous substances, posing risks to both the environment and human health [78]. Table 4 specifies the hazardous substances that may be generated during the production, processing and use of waste plastic-modified asphalt and the risks they pose.

A primary concern is the potential release of polycyclic aromatic hydrocarbons (PAHs) during the production of waste plastic-modified asphalt [79]. PAHs are a class of organic compounds known to be carcinogenic, mutagenic, and toxic to a wide range of living organisms. These compounds can be present in the asphalt binder itself or generated through the thermal degradation of plastic additives during mixing and production processes [80]. For example, research has shown that PS particles can contain high levels of PAHs and have a high adsorption capacity, indicating that PS is likely to release PAHs during high-temperature processing [81]. The volatilization and emission of PAHs can occur at various stages of the waste plastic-modified asphalt production, including the initial heating and melting of the plastic, the blending with the asphalt binder, and the subsequent storage and transportation of the final product [82]. The elevated temperatures involved in these processes can promote the release of PAHs, which may be emitted into the surrounding air, potentially contaminating the workplace environment and nearby communities.

Additionally, the thermal degradation of certain types of waste plastics can lead to the release of other hazardous substances, such as VOCs and chlorinated compounds [83,84,86]. While polyolefin plastics, such as PE and PP, are relatively safer, they can still release toxic VOCs, such as benzene, toluene, and ethylbenzene, if the temperature is not properly controlled during their incorporation into the asphalt [85]. Moreover, PVC can release substantial amounts of HCl and other chlorinated gases when exposed to high temperatures, posing risks to both equipment and the environment [87].

Furthermore, the long-term use of wasted plastics in asphalt can alter its physical properties and potentially release harmful substances during the degradation process. Commonly used plastics produce two greenhouse gases, methane and ethylene, when exposed to ambient solar radiation [88]. Certain plastic types are more susceptible to chemical degradation under high temperatures or UV exposure. This degradation can release not only monomers but also additives, such as plasticizers, stabilizers, and flame retardants, which may include harmful chemicals like phthalates and bisphenol A [89,90]. In addition, studies have shown that 100% of plastic samples contain detectable levels of heavy metals [91]. Throughout the lifecycle of modified asphalt pavement, heavy metals may leach from the plastic due to environmental influences [92].

To mitigate the risks associated with toxic emissions during the production of waste plastic-modified asphalt, a multifaceted approach is essential. This includes implementing robust engineering controls, developing comprehensive environmental monitoring and risk assessment protocols, and adopting stringent occupational health and safety standards [93]. Additionally, further research is needed to develop and evaluate the effectiveness of anti-aging methods and additives that can reduce the release of toxic substances during the production and service life of waste plastic-modified asphalt [94].

### 4.2. Microplastic Generation

The incorporation of waste plastics into asphalt pavement systems not only raises concerns regarding the potential release of toxic emissions during production but also leads to the generation of microplastics during the service life of the road. Microplastics are defined as plastic particles smaller than 5 mm [95]. Degraded microplastics in modified asphalt typically exhibit irregular, fragmented, or fibrous morphologies, with fibers being predominant [96]. The persistence, ubiquity, and potential harm of microplastics to ecosystems and human health make them a growing environmental concern.

**Figure 3:**
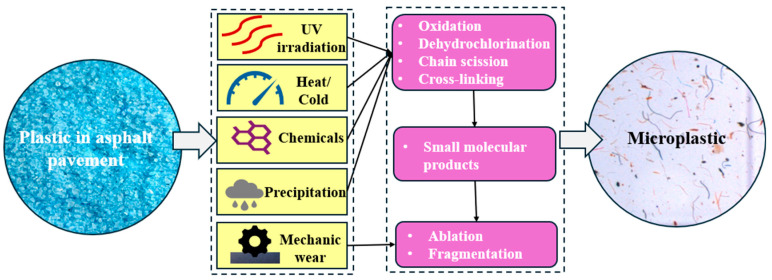
Formation of microplastics in waste plastic-modified asphalt pavements.

Microplastics produced from waste plastics in asphalt pavements primarily arise through abiotic degradation. Continuous traffic loading, exposure to environmental factors, and the presence of chemicals contribute to the breakdown of plastic particles into smaller fragments. The specific process of microplastic generation in waste plastic-modified asphalt pavements is illustrated in Figure 3.

One of the primary mechanisms for microplastic generation in asphalt pavements is mechanical wear caused by repetitive traffic loading and the abrasion between vehicle tires and the road surface [97]. The high shear forces and compressive stresses experienced by the pavement over its service life can gradually degrade the waste plastic particles, resulting in the release of microplastics into the surrounding environment [98]. Smyth et al. [99] demonstrated that pavement wear is a significant source of microplastics in stormwater runoff.

In addition to mechanical wear, the weathering of asphalt pavement contributes to microplastic generation [100]. Exposure to sunlight, precipitation, temperature fluctuations, and other environmental factors can degrade the plastic components, leading to fragmentation and the release of microplastics [101]. Ultraviolet (UV) radiation from sunlight, in particular, can cause the photodegradation of certain types of plastics, such as PE and PP, leading to the formation of smaller plastic particles [102].

Furthermore, the chemical degradation of the waste plastics within the asphalt pavement can also result in the generation of microplastics [100]. The high temperatures and chemical interactions experienced during the production and application of the waste plastic-modified asphalt can lead to the gradual breakdown of the plastic polymers, resulting in the release of microplastic particles. For certain plastic types, such as PS, elevated temperatures can induce thermal decomposition, leading to chain scission in the polymer backbone and the formation of lower molecular weight fragments, which increases susceptibility to further chemical degradation and fragmentation [103].

Microplastics generated in asphalt are predominantly in the size range of 1–500 µm, with their precise dimensions influenced by factors such as the type of plastic, environmental exposure conditions, and duration of exposure. For instance, PET and PS are more susceptible to degradation and fragmentation compared to other plastics [97]. Furthermore, the release quantity of microplastics in asphalt is influenced by a combination of factors, including the type, size distribution, and content of the plastic, as well as the method of its incorporation into asphalt, such as excessive or insufficient plastic dosage, which can increase the generation of microplastics [104].

This research is essential for enhancing our understanding of the mechanisms, size, and quantity of microplastic generation in waste plastic-modified asphalt pavements. Additionally, innovative mitigation strategies and assessments of long-term environmental and health implications are crucial. By proactively addressing the challenges of microplastic generation, the road construction industry can promote sustainable and responsible use of waste plastics in asphalt pavements, balancing economic and environmental benefits with safeguards for human health and the environment.

### 4.3. Ecological Impacts

**Figure 4:**
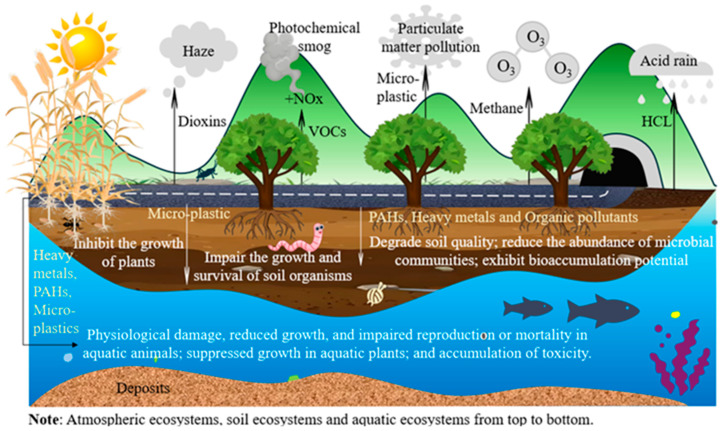
The ecological impact of utilizing waste plastics in asphalt pavements.

The use of waste plastic-modified asphalt pavements poses significant and irreversible threats to the ecological environment through various pathways of contamination, as illustrated in Figure 4. The release of toxic emissions, the generation of microplastics, and the long-term degradation of the pavement materials can have far-reaching consequences for air quality, soil health, and aquatic ecosystems.

During the high-temperature production and application of the waste plastic-modified asphalt, the volatile release of harmful substances from the waste plastics can compromise air quality and the atmospheric environment. For example, the release of HCl can lead to the formation of acid rain, which can acidify water bodies, soils, and vegetation [105]. Dioxins can exacerbate the formation of haze [106]; methane can exacerbate ozone generation [107]. Additionally, the reaction of VOCs, such as benzene, toluene, and ethylbenzene with nitrogen oxides (NOx) under sunlight can form photochemical smog, further impairing air quality [108]. Furthermore, some microplastics resulting from the degradation of asphalt pavements may become airborne, potentially contributing to overall particulate matter pollution [6].

Conversely, some studies have shown that the incorporation of waste plastics improves the atmospheric environment. For example, lifecycle assessment (LCA) indicates that the combination of PET and reclaimed asphalt pavement can reduce carbon dioxide equivalent (CO2e) emissions by up to 26.2% [109]. In addition, although the environmental benefits of reprocessed plastic-modified asphalt decrease with increasing mixing temperature, plastic outperforms competing alternatives (e.g., reducing carbon monoxide emissions) at lower temperatures and an optimal asphalt content of 5.3% [110].

The long-term use of waste plastics in asphalt pavements can also lead to the release of persistent organic pollutants, such as PAHs and microplastics, during the degradation process [111]. PAHs are typical contaminants of soils, causing changes in particle size, porosity and water holding capacity and adversely affecting the diversity or populations of microorganisms. Significant changes in permeability, volume and plasticity also occur, leading to reduced quality of contaminated soils [112]. PAHs impact soil permeability through multiple mechanisms, including the physical obstruction of soil pores by high-molecular-weight compounds, alterations to soil structure through interactions with organic matter and clay particles, suppression of microbial activity that reduces biopore formation, and modifications to soil surface chemical properties [113,114,115]. Microplastics cause damage to soil ecosystems by altering the physicochemical properties of soil texture and structure [116], reducing the abundance and diversity of bacterial communities and inhibiting the biodegradation of exogenous substances in the soil [117], jeopardizing the growth and survival of soil fauna, such as earthworms [118], and strongly affecting the growth of above- and below-ground parts of plants, such as wheat [119]. In addition, microplastics, as carriers of heavy metals and organic pollutants, will further exacerbate soil pollution [120]. For example, phthalates persist in agricultural soils and exhibit bioaccumulation potential due to their high toxicity to soil organisms [121].

The contamination of water bodies is another significant concern associated with the use of waste plastics in asphalt pavements. Stormwater runoff from the road surfaces can carry a variety of pollutants, including heavy metals, PAHs, and microplastics, into nearby streams, rivers, and lakes [122]. Research has shown that in aquatic ecosystems, microplastics can be ingested by plankton, fish, and other aquatic organisms, leading to physiological impairments such as inflammation, oxidative stress, endocrine disruption, and reduced growth and reproduction [123,124]. In severe cases, microplastics can obstruct the respiratory tracts of aquatic organisms, leading to asphyxiation [125] or causing starvation due to a false sense of fullness as microplastics occupy stomach volume and reduce food intake [126]. Additionally, the additives present in waste plastics can release potentially toxic chemicals into the water, further damaging the aquatic ecosystem [127], for instance, by inhibiting the growth of aquatic plants such as microalgae [128]. These contaminants can bioaccumulate in aquatic organisms, leading to the biomagnification of toxins through the food chain, ultimately affecting the overall health and biodiversity of aquatic ecosystems.

The ecological contamination caused by the use of waste plastics in asphalt pavements can also have indirect consequences for the broader ecosystem [129]. The disruption of soil and water ecosystems can lead to habitat loss and the decline in sensitive species, ultimately impacting the overall biodiversity and the resilience of the local environment. The cascading effects of ecological contamination can be far-reaching, potentially compromising the delivery of important ecosystem services, such as nutrient cycling, water purification, and climate regulation.

### 4.4. Human Health Risk

The use of waste plastic-modified asphalt pavements raises substantial concerns regarding potential human health risks. Specifically, workers engaged in the production, application, and maintenance of these pavements encounter heightened risks from direct exposure [130,131].

One pathway of direct exposure is through the inhalation of hazardous substances. During the production and application processes, particularly at elevated temperatures, these materials emit a range of VOCs and PAHs into the atmosphere [132]. Studies also identified HCl as a common emission from asphalt mixtures containing waste plastics [133]. Fumes from asphalt mixtures containing waste plastics demonstrate a positive correlation between PAHs and damage to human DNA [134]; inhalation of particulate-bound PAHs further elevates the risk of lung cancer [135]. Additionally, the reaction of these VOCs with NOx under sunlight can result in the formation of photochemical smog, which increases the risk of cancer, particularly in individuals with cardiopulmonary diseases [136]. Furthermore, exposure to HCl can irritate the upper respiratory tract and may lead to respiratory infections [137].

Another pathway of direct exposure occurs through dermal contact with hazardous substances. Short-term skin exposure may result in minor health issues, such as skin infections. The incorporation of PE and PP into asphalt mixtures significantly increases the concentration of aldehydes and resinous acids. Workers exposed to these mixtures have reported symptoms including eye irritation, throat inflammation, and skin infections [138]. Additionally, short-term exposure to VOCs may manifest as symptoms such as dizziness, headaches, and diarrhea [139]. Prolonged dermal exposure can result in severe health complications, including an increased risk of cancer [140]. For example, VOCs can accumulate in adipose tissue, potentially leading to blood disorders and liver disease [141,142].

Furthermore, the degradation of waste plastic-modified asphalt pavements releases microplastics along with adsorbed organic pollutants, heavy metals, and potentially toxic additives into water bodies, thereby posing indirect risks to human health through drinking water and the food chain [143,144]. Drinking water contaminated with microplastics may induce physiological stress and damage, apoptosis, inflammation, and immune responses [145]. Contaminants can be taken up by plants (e.g., vegetables) and aquatic organisms, subsequently entering the food chain [146,147]. As apex consumers, humans may ultimately intake these harmful substances through bioaccumulation [148], posing risks to human health, including endocrine disruption and developmental or reproductive issues [149,150].

The severity of these health effects largely depends on the concentration of harmful substances entering the human body. Therefore, this study establishes threshold values for key hazardous substances to distinguish safe exposure limits from levels posing health risks (as shown in Table 5). This approach quantifies risks and incorporates safety factors to account for individual susceptibility and uncertainty. Furthermore, these thresholds are aligned with international and national regulatory requirements, providing actionable standards for routine emission control and emergency response to ensure public health protection and facilitate informed decision-making.

## 5. Mitigation and Sustainable Solutions

**Figure 5:**
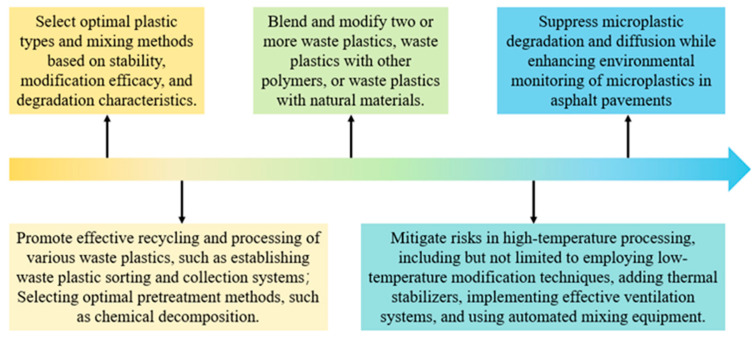
Challenges and responses to the application of waste plastic modified asphalt.

Incorporating waste plastics into asphalt modification offers significant benefits, but the associated environmental and health challenges must be addressed. This study investigates challenges in applying waste plastic-modified asphalt, proposing practical solutions as presented in Figure 5.

### 5.1. Optimization of Waste Plastic Types and Processing Methods

The selection of waste plastic types is critical, as different plastics exhibit distinct performance characteristics and environmental impacts. Appropriate choices can mitigate adverse environmental and performance impacts. A recent study ranked 31 recycled plastic sources based on eight criteria, identifying LDPE and LLDPE as the most suitable for asphalt modification due to their thermal stability and performance enhancement [156]. In contrast, PS incorporation reduces modified asphalt performance and increases microplastic generation. Careful selection of plastics improves modified asphalt performance while minimizing microplastic generation and hazardous emissions during degradation.

Moreover, the blending method also significantly influences environmental impact. The two primary methods are dry and wet processes. While the wet process is more common, it has higher energy consumption and promotes microplastic degradation. Wet-blended plastics are more likely to wear away in the thin surface layer, whereas dry-blended plastics are typically embedded deeper, reducing direct exposure to vehicle abrasion [76]. Thus, the dry blending method is preferable for environmental sustainability.

### 5.2. Enhancement of Recycling and Pre-Treatment Processes for Waste Plastics

Effective recycling and reuse of waste plastics are critical for enhancing asphalt mixing efficiency and minimizing the leaching of hazardous substances. Robust policy support and a comprehensive waste plastic segregation system are necessary to standardize recycling processes and prevent low-quality plastics from entering asphalt mixtures. Enhancing the quality and stability of waste plastics can reduce degradation rates and hazardous substance release. Increased investment in research and development of recycling technologies is also needed to drive innovation.

Pre-treatment methods significantly affect the compatibility of plastics with asphalt. Current studies primarily utilize physical methods such as cutting and grinding, which improve compatibility but may hinder mixing homogeneity [157,158,159]. Researchers have proposed chemical methods, such as grafting HDPE with glycidyl methacrylate, to enhance miscibility [160]. Additionally, irradiation has been employed to form strong chemical bonds between polymer modifiers and asphalt [161]. Specialized processing methods, such as glycolysis for PET, are used for specific plastics [162].

In summary, a comprehensive approach combining effective recycling strategies with enhanced pre-treatment processes is crucial for developing waste plastic-modified asphalt [125]. Improved sorting and processing can enhance plastic quality, compatibility, and mechanical properties while reducing environmental and health risks.

### 5.3. Compatibilized Blending for Optimization

Research indicates that a single modifier may not optimally address issues like the instability of PE-modified asphalt or the sensitivity of PET-modified asphalt to environmental conditions [163]. Consequently, studies are increasingly focused on combining waste plastics with other materials to improve modified asphalt performance. Blending multiple plastics, such as PE with EVA, can enhance stability and compatibility [164]. Additionally, incorporating materials like phosphate monoalkoxy titanate can improve aging and rutting resistance in PS-modified asphalt [165]. Incorporating nanomaterials with plastics in asphalt modification can enhance pavement performance [166].

Innovations also include combining natural materials with waste plastics, such as plant fibers, which can reduce temperature sensitivity [167]. Fillers like diatomaceous earth and slaked lime can enhance durability and performance [168]. Seashell powder can enhance the high-temperature stability, water resistance, and stiffness of asphalt [169]. Additionally, the integration of other industrial byproducts with modified asphalt has been investigated. Zhang et al. [170]. utilized phosphogypsum for asphalt modification, demonstrating that it significantly enhances the rheological properties and aging resistance of asphalt. While compatibilized blending can optimize performance, selecting appropriate compatibilizers tailored to specific waste plastics and application conditions is essential [171].

### 5.4. Risk Control During High-Temperature Processing

High-temperature processing during the production and construction of waste plastic-modified asphalt can release harmful gases and particulates. To manage these risks, it is crucial to improve preparation technology and processing methods by optimizing temperature control and reaction times [172]. Techniques such as adding antioxidants or heat stabilizers can help mitigate degradation at elevated temperatures [173,174]. Employing staged warming techniques can also minimize thermal decomposition and microplastic formation [175].

Furthermore, effective ventilation systems at production and construction sites are vital for safeguarding worker health and minimizing environmental impact. These systems should include local exhaust systems and general ventilation to prevent gas accumulation. Installing gas capture devices and using automated mixing equipment, along with providing protective gear for workers, further reduces health risks.

### 5.5. Degradation and Spread Control of Microplastics

To mitigate microplastic generation and environmental impact, several strategies have been explored. Incorporating dynamic crosslinks into polymer structures can enhance asphalt adaptability and prevent microplastic degradation [162]. Surface treatments, such as protective coatings, can further reduce degradation rates. Research suggests that permeable pavements may effectively retain microplastics, serving as a valuable tool for pollution mitigation [176].

However, complete prevention of microplastic degradation is not feasible, making spread control essential for environmental protection. Installing filtration devices in drainage systems can capture microplastic particles, preventing their entry into soil and water bodies. Strengthening environmental monitoring of microplastics in asphalt pavements is also crucial, as other hazardous substances may migrate alongside degraded plastics [177]. Challenges in detecting microplastics include limited technology for detecting small particles and the need for standardized equipment to simulate road traffic [178]. Regular monitoring of microplastic concentrations in stormwater runoff and soil will provide valuable data on the environmental impact of using waste plastics.

## 6. Conclusions and Future Perspectives

The incorporation of waste plastics into asphalt pavements involves a balance between engineering benefits and environmental challenges. This innovative approach not only offers a viable solution for plastic waste management but also enhances the mechanical performance of asphalt. However, it is essential to acknowledge the environmental implications associated with these practices. The main findings of this review are summarized as follows:

(1) Waste plastics generally improve asphalt’s high-temperature stability and fatigue resistance, with LDPE and EVA exhibiting superior overall performance. However, the efficacy of plastics varies by type: PVC may release toxic emissions, while PS and PET are prone to generating microplastics. Compatibility issues and durability reductions remain challenges, necessitating tailored selection based on application scenarios.

(2) The wet process enhances asphalt–plastic integration but demands high energy and specialized equipment, with increased microplastic degradation risks. The dry process is simpler and more cost-effective but may compromise uniformity and long-term durability. Selection should align with plastic properties and project requirements.

(3) Toxic emissions (PAHs, VOCs, HCl) during production threaten air quality, while microplastics generated via mechanical wear and weathering contaminate soil and water ecosystems. Heavy metals and additives leaching from plastics further exacerbate ecological harm. Human health risks include direct exposure (inhalation, dermal contact) for workers and indirect risks via the food chain, emphasizing the need for strict exposure controls.

(4) Sustainable adoption requires several strategies: prioritizing low-risk plastics and optimizing processing; advancing pre-treatment and compatibilization to improve dispersion and reduce degradation; controlling high-temperature processing to minimize emissions; and implementing microplastic retention measures and monitoring protocols.

In summary, waste plastic-modified asphalt offers a promising solution to the plastic crisis and infrastructure needs, but its sustainable deployment hinges on addressing risks through interdisciplinary strategies—combining material science, environmental engineering, and policy support.

## Figures and Tables

**Table 1:** Basic properties of waste plastics.

Types	Melting Point (°C)	Main Sources	Mechanical Properties	Environmental Considerations	Ref
LDPE	110–120	Plastic wraps and bags; soft drink and water bottles; trays and containers	Low stiffness and strength; high ductility	Generally low toxicity; limited recycling options.	[25,26]
HDPE	135	Plastic bottles and packaging; plastic chairs/stools; toys; playground equipment	Moderate stiffness; low strength and ductility	Widely recyclable and has a low environmental impact.	[27,28]
PP	160–170	Straws; furniture; reusable plastic containers; plastic moldings	Medium stiffness; low strength; high ductility; chemical resistance	Less commonly recycled, leading to potential waste issues	[29,30]
PS	230–240	Disposable utensils; CD and DVD casings; carry-out containers; foam beverage cups	High stiffness and strength; very low ductility	Often not recyclable; can break into microplastics	[31,32]
PVC	160–210	Pipes; construction materials; sign boards	High stiffness; medium strength and ductility	Potential release of toxic substances.	[33,34]
PET	250	Soft drink and water bottles; food packaging	High stiffness and strength; medium ductility	Highly recyclable; potential microplastic pollution.	[35,36]

(Note: The mechanical properties described in the table are based on mean values of relevant performance metrics established in this study: elastic modulus E = 2 GPa, tensile strength σₜ = 50 MPa, and elongation at break ε = 100%).

**Table 2:** Asphalt modification effects of common waste plastics.

Type	Compatibility (Molecular Structure/ Melting Point)	High-Temperature Stability (Complex Modulus (G *), Phase Angle (δ))	Low-Temperature Crack Resistance (BBR Test Values/ MCI/Brittleness)	Anti-Fatigue Property (Aging Index/ Fatigue Life (Nf))	Ref
LDPE	↑	↑↑	↓	↑	[38,39,40,41]
HDPE	↓	↑↑	↓	↑	[42,43,44,45]
PP	↑	↑↑	↓	↑	[46,47]
PS	↓	↑	↓	↓	[48,49,50]
PVC	↓	↑	↑	↑	[51,52,53]
PET	↓	↑↑	↓	↑	[54,55,56]
EVA	↑↑	↑↑	↑	↑↑	[57,58,59]

(Note: The * in the complex modulus (G) denotes that it is a complex quantity, reflecting the material’s simultaneous elastic and viscous behavior under dynamic loading; “↑” for “mild improvement”, “↑↑” for “significant improvement”, “↓” for “reduction”).

**Table 3:** Comparison of various properties of dry and wet process.

Aspect	Wet Process	Dry Process
Process Description	Waste plastics are first melted and blended with asphalt before being mixed with aggregates.	Waste plastics are first added to hot aggregates and then mixed with asphalt.
Plastic Types	Soluble in specific solvents or compatible with asphalt.	Requires high melting points (>180 °C) and thermal stability.
Performance Outcomes	Extended compatibility and service life; greater resistance to fatigue and permanent plastic deformation; uncertain long-term storage stability.	Improved Marshall stability, ITS values, and TSR; relatively low compatibility and durability
Processing Conditions	Complex and stringent processing procedure; specialized equipment; higher costs.	Simpler processing procedure; general equipment; more cost-effective.
Environmental Impacts	Higher energy consumption; more likely to promote the degradation of microplastics.	Lower energy consumption and microplastics; may generate more dust and airborne particles.

**Table 4:** Potential hazardous substances and associated risks from waste plastic-modified asphalt.

Substance	Specific Compounds	Emissions Volume	Emission Scenarios	Associated Risks	References
PAHs	Pyrene; acenaphthene; phenanthrene; fluorene	0.4–6.7 µg/m^3^	Whole stages of production	Carcinogenic; respiratory irritation	[79,80,81,82]
Volatile Organic Compounds (VOCs)	Benzene; styrene; trichloroethylene; tetrachloroethylene	0.2–4.5 µg/m^3^	High-temperature processing	Air contamination; endocrine disruption	[83,84,85,86]
Chlorinated Compounds	HCl; other chlorinated gases	Massive	Thermal degradation	Damage to instruments and the environment	[87]
Greenhouse Gases	Methane; ethylene	10–5100 pmol g^−1^d^−1^	Exposure to solar radiation	Contributes to climate change	[88]
Plastic Additives	Phthalates; bisphenol A	1–2 mg/L	Long-term use	Endocrine disruption	[89,90]
Heavy Metals	Chromium; copper; lead; nickel; zinc	0.25–0.51 mg/kg	Degradation over lifecycle	Environmental contamination; toxicity	[91,92]

**Table 5:** Benchmark values for key pollutants in waste plastic-modified asphalt.

Pollutant	Exposure Pathway	Health Risks and Regulatory Threshold	Guideline Level	Source
PAHs	Respiratory; dermal; digestible	Carcinogenicity (0.2 µg/L)	MCL	EPA NPDWR [151]
VOCs	Respiratory; Dermal	Hematotoxicity (1.7 µg/m^3^); neurotoxicity (5 mg/m^3^); carcinogenicity (5 µg/m^3^) respiratory irritation (0.1 mg/m^3^)	RfC; MCL	WHO; EPA IRIS; EPA NPDWR [151,152,153]
HCL	Respiratory	Upper-airway irritation (2 mg/m^3^)	AEGL-1	EPA AEGL [154]
Heavy Metals	Digestible	Carcinogenicity (10 µg/L); neurodevelopmental effects (15 µg/L)	Action Level	EPA NPDWR [151]
Plastic Additives	Digestible	Reproductive/developmental toxicity (20 µg/kg); endocrine disruption (50 µg/kg)	RfC	EPA IRIS [153]
Microplastics	Digestible	Physical damage; apoptosis; inflammation; oxidative stress and immune responses (no guideline)	Under review	WHO [155]

(Note: AEGL: Acute Exposure Guideline Level. EPA: Environmental Protection Agency. IRIS: Integrated Risk Information System. MCL: Maximum Contaminant Level. NPDWR: National Primary Drinking Water Regulations RfC: Reference Dose. WHO: World Health Organization.)

## Data Availability

No new data were created or analyzed in this study.
